# Satisfied or Frustrated? A Qualitative Analysis of Need Satisfying and Need Frustrating Experiences of Engaging With Digital Health Technology in Chronic Care

**DOI:** 10.3389/fpubh.2020.623773

**Published:** 2021-01-26

**Authors:** Carolina Wannheden, Terese Stenfors, Andreas Stenling, Ulrica von Thiele Schwarz

**Affiliations:** ^1^Department of Learning, Informatics, Management and Ethics, Karolinska Institutet, Stockholm, Sweden; ^2^Department of Psychology, Umeå University, Umeå, Sweden; ^3^Department of Sport Science and Physical Education, University of Agder, Kristiansand, Norway; ^4^School of Health, Care and Social Welfare, Mälardalen University, Västerås, Sweden

**Keywords:** self-tracking, digital health (eHealth), persuasive technologies, motivation, design, user experience (UX) evaluation, self-determination theory (SDT), self-monitoring devices

## Abstract

**Introduction:** Digital health technologies such as self-monitoring devices and apps are becoming increasingly important as tools to promote healthy habits and support individuals in their self-care. There is still a scarcity of research that builds on motivational theory to better understand the functioning of digital health technologies. The self-determination theory (SDT) is a macro theory of motivation that delineates three basic psychological needs that are linked to different types of motivation and lead to well-being when satisfied and illbeing when frustrated.

**Objective:** To explore how the use of a digital tool for self-monitoring and communication with healthcare satisfies or frustrates basic psychological needs across four spheres of user experience: interface, task, behavior, and life.

**Methods:** The study was conducted in a Swedish primary care setting with individuals who participated in a pilot study of a digital health intervention for self-monitoring in chronic care management. Data from a follow-up survey with participants 7 months after recruitment were analyzed using a thematic approach mixing inductive and deductive analysis. The unit of analysis is based on a total of 642 individual answers to seven open-ended questions, from 121 respondents.

**Results:** The analysis identified positive and negative influences of self-monitoring and digital communication with healthcare on all three psychological needs. Three main findings are that: (1) data covered all four spheres of user experiences, but most user experiences concerned the behavior and task spheres; (2) satisfaction and frustration of competence needs was more prominent than influences on other needs; (3) the same experience may be perceived as both need frustrating and need satisfying, which suggests a tension that reflects individual differences.

**Conclusion:** Designers of digital health technologies need to take into account basic psychological needs within all spheres of user experience, from interface to life in general. Because some features may be simultaneously experienced as satisfying and frustrating by different users, these types of tools need to be flexible to accommodate for variation of user experiences. Careful design considerations that take motivational theory into account would contribute to the transformation of care for individuals with chronic conditions.

## Introduction

For individuals with chronic health conditions such as high blood pressure or diabetes, self-management is inescapable. What they do throughout the day will have an impact, positive or negative, on their condition (1). Good health outcomes are dependent on self-management of symptoms as well as healthy behaviors such as diet, physical exercise, and sleep (2). The acknowledgment of the patients' knowledge, experience, and influence on their own care has transformed chronic care management, where self-care has been predicted to become the new principal source of care for an increasing number of individuals who have the ability and necessary support to engage in self-care (3).

To promote healthy behaviors, digital health technologies such as self-monitoring devices and apps are becoming increasingly important by facilitating tasks such as identifying symptoms, planning treatment, monitoring key health parameters, and monitoring progress and treatment effects (4, 5). Thus, these types of tools have the potential to support individuals with chronic conditions in their self-care. This assumes that the technology is used, which in turn builds on users being willing to engage with the technology, that is, that they are motivated to use it (6). While theories of acceptance and use of technologies [see e.g., (7)] have long been a concern for designers of digital health technologies, our understanding thereof is still limited (8). Motivation has only recently been taken into account in design considerations for digital technologies. The interest in motivation is visible in fields such as persuasive technology (9), which deals with technologies that are designed to support healthy behavior change. Persuasive technologies have been classified into gamification, quantified-self, and social networking (10). Substantial effort has been put into describing the so-called motivational affordances offered to users, which refers to the properties of a technology that determine if it supports users' motivational needs (8). In the area of health and wellness, some of the most common strategies employed by persuasive technology interventions involve self-monitoring, performance analysis, exercise guidance, rewards, feedback, social recognition, social comparison, watching others, and self-presentation (9, 11).

Yet, the research that builds on motivational theory to better understand the functioning of digital health technologies is still limited (10, 12). For example, a review of the literature on technologies aiming to aid and motivate individuals to engage in healthy life habits concluded that more than half of the studies included in their review were not informed by any motivational theory, and most of those that referred to a motivational theory only mentioned it without specifying how it informed the study (9). Thus, the underlying psychological processes that would explain why individuals may perceive a certain feature as motivating are largely unexplored (13).

Self-determination theory (SDT) is a macro theory of motivation that has been used extensively in over four decades to explain human motivation in various domains (14), including behavior change and health behaviors (15, 16). The theory distinguishes between different types of motivation and delineates three basic psychological needs that explain intrinsic motivation (and autonomous types of extrinsic motivation): autonomy, competence, and relatedness (17). When these needs are satisfied, they are inherently rewarding, lead to psychological well-being and flourishment. When frustrated, they lead to negative experiences, such as passivity, illbeing, and defensiveness (18–21). The three basic psychological needs are assumed to be universal, and the satisfaction of the needs is crucial for well-being and functioning in all contexts and across the lifespan (14).

While there are a number of empirical studies that have explored *interpersonal* need support across various fields [e.g., (15, 22, 23)], need support by other means, such as digital health technologies, is less explored. In the domain of human-computer interaction, it has been shown that playing games is a highly intrinsically motivated behavior (24), considered to satisfy all three psychological needs (25). Thus, SDT has in recent years more commonly also been used as a theoretical frame to study the motivational potential of incorporating gameful elements and processes into information systems and services in other contexts (i.e., gamification), where digital health technologies form one of the largest application areas, preceded by the domain of education and learning (13).

The most studied basic psychological need, both in general and in relation to human-computer interaction and digital health technologies, is *autonomy*, which describes the sense of willingness and volition that stems from activities that are in accordance with one's personal goals and values (14). Studies on gaming have indicated that autonomy, for example, may be supported by offering options for tailored support (26). Tailoring strategies involve personalization (making the information more meaningful to the recipient), feedback (presenting individuals with information about themselves), and content matching (directing messages to individuals' needs in relation to aspired behaviors) (27). Tailoring technologies may also facilitate autonomy by allowing the individual to set individual goals, or by removing obstacles to goal pursuit (28).

The second need is *competence*, which is the psychological need for feeling capable and efficient. It is the psychological need that most consistently has been shown to predict engagement in physical exercise (29). Digital health functions, features and content such as the level of challenge and the degree of feedback on behavior or learning opportunities are factors that may satisfy or frustrate the need for competence. For example, the level of challenge (not too easy and not too difficult) was the most frequently reported design feature explaining youth's motivation to engage in digital health lifestyle games in a systematic review of the literature (28). Positive feedback was another feature found to foster engagement, possibly by making the individual feel capable and efficient (26).

The third basic psychological need is *relatedness*, which describes the need to belong and feel connected to others. In this, it explains why social interaction in itself may not always be related to well-being: social interaction can satisfy or frustrate people's need for relatedness (10). Research on the relationship between need support for relatedness and social media technology, for example, indicate that features that facilitate active direct interaction over passive interaction may be a more need satisfying design choice (30), that recognition from others is important (13), and that in games to promote healthy life habits, identification with characters may increase engagement (28). On the other hand, relatedness may not always be an issue, such as when someone prefers to perform the activity solitarily (such as some prefer to exercise) (29).

To date, there are few studies that have investigated both need satisfaction and need frustration, although the very same features that satisfy a need may also frustrate one (e.g., feeling a sense of belonging or feeling left out) (31, 32). An exception in the health domain is a study that focused on fitness apps using self-monitoring, rewards, and social recognition features (31). The study showed that whereas self-monitoring increased competence satisfaction and decreased competence frustration, the incorporation of rewards and social recognition features concurrently satisfied the need for competence and increased competence frustration (31). A model that allows for the exploration and understanding of simultaneous experiences of need satisfaction and need frustration in relation to digital technology use is the METUX (Motivation, Engagement and Thriving in User Experience) model (12), which is based on SDT to understand the impact of digital technology on motivation, engagement, and well-being. A recent review on ethical issues related to digital well-being suggested that this is the most comprehensive framework to date for evaluating digital well-being (33).

The METUX model outlines the three basic psychological needs as the mediators between technology design and user experience (12). After a technology has been adopted, the model describes four spheres of user experience where psychological needs should be considered: (1) in the interaction with the technology interface, (2) in the engagement with technology-enabled tasks such as self-monitoring, (3) in technology-supported behaviors related to healthy habits (e.g., physical exercise, sleep hygiene etc.), and (4), in an individual's life in general. The model provides a lens through which technologies' impact on user experience can be understood, that is, how technology can satisfy or frustrate the basic psychological needs of users at different levels. This, in turn, can explain paradoxical observations, for example that self-monitoring and self-care can both empower and disempower patients (34).

Moreover, satisfaction of needs in one sphere may unintentionally frustrate needs in another sphere (31, 35). For example, the gaming industry provides illustrative examples of how technology can be need supportive in the interface or task domain but at the same time frustrate healthy life habits (32, 36), and healthy lifestyle technologies may be effective in the sense that it is satisfying to interact with their interface but still not supportive of needs related to the behaviors the app was developed to affect (37). Yet, these cross-sphere implications of need satisfaction have largely been overlooked (12, 38). Thus, while there are studies that explore motivational aspects of the user interface, there are few studies that look at the combined motivational impact across interface, tasks, behaviors, and life in general. Conceptually, it has been suggested that some needs may be more vital to satisfy in some spheres than others (e.g., it may be more important that the interface satisfies the need for autonomy than relatedness, whereas relatedness satisfaction is vital in the life sphere) (12). However, how the different needs are satisfied across the different spheres remains to be empirically investigated. Thus, the aim of this study was to explore how the use of a digital tool for self-monitoring and communication with healthcare satisfies or frustrates basic psychological needs across four spheres of user experience: interface, task, behavior, and life. This knowledge can be useful to inform designers about the potential motivational impacts of digital health technology use, which may in turn guide the choice of design functions, features, and content.

## Methods

The study was conducted in a Swedish primary care setting with individuals who participated in a pilot study of a digital health intervention for self-monitoring and digital communication with healthcare staff in chronic care management. The digital health service that was used was a Swedish adaptation of a service that was originally developed at Dartmouth-Hitchcock Health for managing chronic conditions (39). It consisted of monitoring devices (activity tracker bracelet, blood pressure cuff, scale) and a smartphone application. The smartphone application had three core features: health data tracking through sensors and manual input; a personal profile for documenting health goals, preferences and social data; and secure communication to connect with healthcare staff through chat and video. The application enabled automatic sharing of patients' self-monitored data (activity, sleep, and monitoring of selected health parameters) with healthcare staff. In case of potentially serious deviations, alerts were communicated to both patients and healthcare staff. Motivational features consisted of automatically communicated clinical information and suggestions, nudges, and support to patients. Further, linguistic variation was used to personalize communication, and the choice of color and language aimed at supporting positive behavior change, enjoyment, as well as reducing stress and anxiety (39). Further details on the original development and implementation of this technology in a US setting are described here (39).

### Recruitment and Data Collection

Participants were recruited through the primary care organization among patients over 18 years of age diagnosed with hypertension, chronic heart failure, or mental health conditions (including reaction to severe stress and adjustment disorders, insomnia, anxiety disorders, and depressive disorders). Further inclusion criteria for participating in the intervention study were that participants had a smartphone, an email account, and were able to communicate in Swedish. Identified patients were invited to a group meeting at the primary care center where they were equipped with all necessary devices. An activity tracker bracelet (tracking steps and sleep) was provided to all participants. Individuals with hypertension were also equipped with a blood pressure cuff and some (in particular patients with heart failure) were equipped with a scale. All monitoring devices could be paired with the mobile application using Bluetooth. One of the researchers participated in these group meetings where she informed about the research study and collected contact details of individuals who volunteered to be contacted with an invitation to participate in pre- and post-intervention surveys. Ethical approval for the study was granted by the Regional Ethical Review Board of Stockholm in 2018 (reg nr. 2018-1717-32).

The present study uses data from open-ended questions in the post-intervention survey that was distributed to participants of the intervention study 7 months after recruitment was completed. All respondents provided their informed consent to participate in the study. A total of 134 individuals responded (after two reminders), out of which 122 provided answers to open-ended questions in the survey. The unit of analysis is based on a total of 642 individual answers. The spread in number of answers per individual was median 6 (IQR 4-7). The questions and their individual response rates are shown in Table 1. Participant demographics are described in Table 2. The majority of respondents (52%) were female, over 60 years of age (51%), and used the digital health technology to manage hypertension (68%). A considerable proportion (28%) of the participants reported that they used the digital health technology as a support for more than one health condition. Some (4%) did not know why they used the digital health technology, or used it for other reasons than hypertension, heart failure or mental illness (7%). The most frequently used functionality (86%) was the activity tracker bracelet for monitoring the number of steps and sleep, and the majority of respondents (57%) declared to interact with the digital health technology at least once per day.

**Table 1:** Open questions with individual response rates.

**#**	**Question**	**Responses, *n* (%)^**a**^**	**Words, *n* (%)^**b**^**
1	Describe in your own words how often and how you used [eHealth tool]:	113 (93%)	1571 (22%)
2	Did your use of [eHealth tool] lead to any changes in your treatment? If yes, in what way?	89 (74%)	773 (11%)
3	What were the challenges with using [eHealth tool]?	93 (77%)	1012 (14%)
4	Describe in your own words how the use of [eHealth tool] has influenced how you take care of your health:	93 (77%)	1197 (17%)
5	Describe in your own words how the use of [eHealth tool] has influenced your consumption of healthcare services:	75 (62%)	899 (12%)
6	What consequences (positive and/or negative) did the use of eHealth tool] have for you?	91 (75%)	1059 (15%)
7	What have you learned from using [eHealth tool]?	88 (73%)	716 (10%)

For each question, we also indicate the accumulated word count from all individual responses combined.

a Percent of total number of respondents (N = 122);

b *Percent of total number of words (N = 7,227)*.

**Table 2:** Demographic details of respondents (*N* = 122).

**Variable**	**Frequency**	**Percent**
**Gender**		
Male	46	38%
Female	64	52%
Missing^a^	12	10%
**Age in years**		
20–29	3	2%
30–39	6	5%
40–49	8	7%
50–59	31	25%
60–69	44	36%
70 or older	18	15%
Missing^a^	12	10%
**DHT used for**^**b**^		
Hypertension	83	68%
Heart failure	10	8%
Mental illness	24	20%
Don't know/Other	13	11%
Missing^c^	4	3%
**Use of functionalities**		
Activity tracker bracelet	105	86%
Blood pressure cuff	95	78%
Scale	12	10%
Chat	42	34%
Phone/Video	9	7%
**Frequency of interaction with DHT**		
At least once per day	69	57%
At least once per week	36	29%
At least once per month	7	6%
Less than once per month	8	7%
Missing^c^	2	2%

DHT, Digital health technology;

a Age and gender were only reported in the pre-intervention questionnaire; missing values for respondents who only filled in the post-intervention questionnaire;

b Numbers add up to more than 100% in this category because some used the DHT for more than one diagnosis;

c *Missing values because it was not mandatory to respond*.

### Analysis

The data were analyzed in several steps mixing an inductive and deductive thematic approach (40). The open-ended responses were labeled with a respondent ID and question number and transferred to a mind mapping software (FreeMind, licensed under the GNU General Public License Version 2) for categorization. The first author read through all the text (7,227 words) and created initial codes inductively, within each of the open questions separately. In the next step of the analysis, overlapping categories across the different questions were merged. Thereafter, a deductive coding framework was applied, based on the satisfaction or frustration of the three basic psychological needs, as described in (18) (Table 3). This preliminary analysis was presented to the co-authors and discussed in frequent meetings in which categories were merged, moved, or deleted until negotiated consensus was reached. After this, the spheres of user experience as described in (12) (Table 3) were applied to the dataset resulting in another layer of deductive coding. This analytic step was conducted by the first author in close collaboration with the co-authors. All co-authors are full time researchers with training in qualitative research methods.

**Table 3:** Coding scheme defining the concepts of needs satisfaction and frustration, based on (18), and spheres of user experience, based on (12).

**Concept**	**Definition**
Autonomy satisfaction	Experiences reflecting willingness and volition with respect to using the digital health technology and/or engaging in behaviors supported by the digital health technology. Satisfaction is characterized by “a sense of integrity as when one's actions, thoughts, and feelings are self-endorsed and authentic.”
Autonomy frustration	Experiences reflecting pressure or conflict, such as being pushed in an unwanted direction with respect to using the digital health technology and/or engaging in behaviors supported by the digital health technology.
Competence satisfaction	Experiences reflecting effectiveness, mastery, and opportunities for using and extending one's skills and expertise with respect to using the digital health technology and/or engaging in behaviors supported by the digital health technology.
Competence frustration	Experiences reflecting “a sense of ineffectiveness or even failure and helplessness” with respect to using the digital health technology and/or engaging in behaviors supported by the digital health technology.
Relatedness satisfaction	Experiences reflecting connectedness, involvement and a feeling of significance in relation to others with respect to using the digital health technology and/or engaging in behaviors supported by the digital health technology.
Relatedness frustration	Experiences reflecting “a sense of social alienation, exclusion, and loneliness” with respect to using the digital health technology and/or engaging in behaviors supported by the digital health technology.
Interface sphere	Experiences relating to the interaction with the digital health technology via its interface.
Task sphere	Experiences relating to engaging with digital health technology specific tasks.
Behavior sphere	Experiences relating to the engagement in behaviors that the digital health technology is intended to support.
Life sphere	Experiences reflecting an individual's overall life, beyond the digital health technology.

The deductive coding steps led to the exclusion of some open-ended question responses that could not be classified based on our coding framework. Experiences that described outcomes, without further explanation of which needs were satisfied or frustrated, were not included in the analysis. Examples are: “*I exercise more,” “I keep a healthier diet,” “My blood sugar was reduced,” “I experienced no effects,” “Stressful to perform self-monitoring.”* We also identified and excluded descriptions of experienced satisfaction of safety and security, which have historically not been considered as basic psychological needs in SDT. Rather, they have been defined as deficit needs or need-substitutes that occur as a response to need frustration (14, 18, 41). The reasoning is that people are quick to desire safety-security when their needs are frustrated or thwarted (i.e., when the self is threatened). Hence, safety and security are primarily considered as outcomes of need frustration rather than basic psychological needs in their own right and therefore not included in our analysis. Further, some expressions that were unclear or too difficult to interpret were excluded. This included expressions like: “*Increased motivation,” “Motivated more steps and thoughts about sleep.”* While some of the excluded responses contribute to evaluate behavioral and health outcomes of the digital health intervention, this was not within the scope of this study.

After exclusion of data, our categorization comprised a total of 312 unique responses, split into 360 descriptive codes. While some qualitative researchers argue against any quantification of qualitative data, we are of the opinion that the display of numerical information makes patterns in our data emerge more clearly (42), without making any suggestions regarding the relative weight or importance of individual themes over others. Thus, in our analysis, we present the number of unique responses that contributed to each of the themes that were identified.

## Results

**Figure 1:**
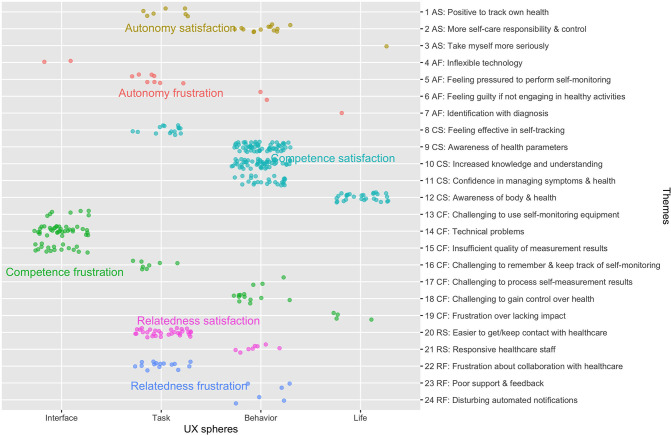
The different themes of need satisfaction and frustration within different spheres of user experience (UX). The dots represent the number of individual codes that contributed to each of the identified themes and user experience spheres. The colored dot fill reflects our classification into autonomy satisfaction (AS), autonomy frustration (AF), competence satisfaction (CS), competence frustration (CF), relatedness satisfaction (RS), and relatedness frustration (RF).

The analysis resulted in the identification of experiences that illustrate satisfaction as well as frustration of the three basic psychological needs, in four spheres of user experience. The results are summarized in Figure 1 and described with illustrative quotes in the sections that follow.

### Autonomy

*Autonomy satisfaction* was identified in respondent descriptions of the ability to take more responsibility in monitoring their own health parameters (task), controlling their self-care (behavior), and taking themselves more seriously (life). For example, the performance of routine monitoring at home was described to lead to more engagement in physical activities and to increase patients' involvement in healthcare. Respondents described how this made them feel more in control and independent, such that the need for healthcare visits and services may decrease. The following quote provides an example of autonomy satisfaction in the task and behavior spheres:

*I could keep track of my blood pressure, I stopped eating medications slowly until my current state in which I am completely free from them, I can say that it was the best thing I've taken part of concerning my health, I could really take responsibility for trying to feel well, and have reached several goals thanks to [eHealth service]; it was really a disappointment for me when this service was taken away* 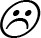 *I feel that I don't have the same level of oversight that I had before* 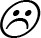 *(ID: 4, question 2)*

*Autonomy frustration* concerned experiences of inflexibility in the technology (interface), feeling pressured to perform self-monitoring (task), feeling guilty if not engaging in health-promoting activities (behavior), and identification with one's diagnosis (life). For example, respondents expressed that they had been asked by healthcare staff to more frequently self-monitor their health parameters, which can be interpreted as external pressure. As one respondent put it, he/she felt that he/she was no longer expected to visit primary care. Some experienced it as time-consuming and challenging to establish a routine for daily monitoring. There was also some frustration caused by message notifications that could not be turned off. One respondent described how he/she had started to feel more guilt when not engaging enough in physical activities. The following quote illustrates how the use of a digital service for self-monitoring and communication with healthcare negatively influenced an individual's overall experience of life:

It feels like I've been marked with psychological illbeing because of a short period of exhaustion disorder and I have not had the chance to change this mark. (ID: 91, question 6)

### Competence

*Competence satisfaction* was identified in descriptions of respondents' feelings of effectiveness in self-monitoring (task), increased awareness, knowledge and understanding about health parameters, as well as confidence in managing symptoms (behavior), and awareness about one's body, health and life in general (life). Effectiveness in self-monitoring was described both in terms of the ability to perform their own monitoring and the increased regularity by which this is done. Some explained that their own monitoring reduced the frequency of care visits, which could also be interpreted as a sign of autonomy satisfaction. The increased awareness was described in terms of specific health parameters (e.g., blood pressure, sleep, and physical activity) or in more general terms. As one respondent put it: “*I have a better understanding of myself and that feels good” (ID: 145, question 5)*. Several aspects of knowledge and understanding were raised: the importance of controlling one's health parameters, the importance and effects of one's behavior, effects of treatment, and the ability to pose new questions and make assessments about symptoms and health. The following quote illustrates how increased knowledge and understanding could be used effectively in collaboration with healthcare staff:

I understood that my blood pressure can vary quite a lot, but because I after a while could prove that my blood pressure was not too high, rather low, the medication could be reduced. My blood pressure was almost always relatively high when it was measured at the primary healthcare center. (ID: 23, question 4)

*Competence frustration* was identified in all spheres of user experience. In the interface sphere, respondents expressed challenges using the self-monitoring equipment, technical problems, as well as insufficient quality of self-monitoring results that hindered them from feeling effective. In the task sphere, it was described as challenging to remember and keep track of self-monitoring. To remember to monitor health parameters was one challenge, another was to remember certain functionalities that had to be activated in order to properly log activities. In the behavior sphere, some described that it was challenging to be confronted with their self-monitoring results and gain control over their health, for example keeping blood pressure on the right level. Finally, in the life sphere, respondents expressed a general frustration over lacking impact of the digital health intervention. The following quote illustrates competence frustration in the behavior sphere:

[What have you learned from using the eHealth service?] – That my blood pressure varies a lot – but not how I should take care of it. I don't know why it varies or what I should do to lower it. I have been prescribed another pill – that's all. (ID: 1, question 7)

### Relatedness

*Relatedness satisfaction* was identified in respondent descriptions of improvements in getting and keeping contact with healthcare staff (tasks) and responsiveness of healthcare staff on patients' reported health observations (behavior). Respondents described that the digital health technology made it easier for them to get in touch with and access primary care. Some also described that they preferred to use the chat functionality when they felt unable to call due to psychological distress. In terms of responsiveness, respondents described that it felt good that healthcare staff had access to their reported health parameters and sometimes commented with supportive feedback. One of them described this as “*a feeling of being ‘surveilled' in a positive sense” (ID: 75, question 6)*.

*Relatedness frustration* concerned challenges in the communication and collaboration with healthcare staff induced by the digital health technology (task). Some described that they did not get answers to the questions they had posed in the chat. Others described challenges in knowing what types of questions they could pose and daring to write about their needs and experiences. Some also described the lack of personal contact by communicating digitally and the desire to meet healthcare staff face-to-face. Frustration was also expressed in relation to the experience of insufficient support and feedback from healthcare staff in one's self-care (behavior). Few expressed that they experienced the feedback that was provided as disturbing or mechanic. As one of the respondents put it:

I experienced it as very negative that what I experience as an organization behind the app took part of my data and made brisk comments like “Well done” because my blood pressure had changed between two measurements. That is unlikely an effort on my side for such a change to occur. That made me not want to use the app after a while. Also, I didn't understand why the selected measurement methods were included to communicate with primary care. I don't feel a need to continuously show my weight and physical activity to the primary care center. (ID: 41, question 1)

## Discussion

This study explored how the use of a digital health technology for self-monitoring and communication with healthcare in chronic care management satisfied or frustrated users' basic psychological needs of autonomy, competence, and relatedness over four spheres of user experience. Three main findings attracted our attention in the analysis and will be discussed below. First, most user experiences concerned the behavior and task spheres, rather than the interface and life spheres. Second, experiences of influences on the need for competence were more prominent than the influence on other needs in our data, both in terms of need satisfaction and need frustration. Third, tensions were revealed between the satisfaction and frustration of all three needs, which may indicate individual variations. Our findings call for increased flexibility in the design and use of motivational affordances in the design of digital health technologies to support self-care of chronic conditions.

### User Experiences Across Four Spheres

Influence on the satisfaction and frustration of basic psychological needs was reflected in four explored spheres of user experience in the METUX model (12), from interactions with the interface and technology-specific tasks to behaviors and life in general. Most of our data, in terms of quantity, concerned the behavior and task spheres of user experience. While all themes in the interface sphere reflected need frustration, experiences of need satisfaction stood out in the behavior and life spheres. Thus, despite reported challenges in the interaction with self-monitoring equipment and a perception of insufficient quality in monitoring results (i.e., challenges related to the perceived usability of the digital health technology), individuals experienced that their awareness of both specific health parameters and their health in general increased. This suggests that the satisfaction or frustration of a psychological need in one sphere does not necessarily predict experiences in other spheres. We identified both satisfaction and frustration of needs in the behavior and task spheres. Some experiences reflected autonomous motivation whereby respondents expressed an identified value or importance of engaging in digital health-supported tasks and behaviors (*identified regulation*). Other experiences reflected controlled motivation that was characterized by individuals feeling pressured by healthcare to perform self-monitoring *(external regulation)* or by feeling guilty if not engaging in healthy activities (*introjected regulation*). These types of controlled motivation are known to have a negative impact on long-term adherence to healthy behavior change (43, 44). Thus, further research is needed to explore long-term effects as well as the relation between the satisfaction and frustration of needs in different spheres of user experience.

It has been suggested that all three psychological needs do not necessarily have to be satisfied at all levels (12). In particular, it has been suggested that relatedness does not need to be satisfied in every interaction with technology and may not be essential in the interaction with an interface (12, 45). In our study, satisfaction and frustration of relatedness was found mainly within the task and behavior spheres, but not in the interface sphere. This supports the claim that satisfaction of some needs may only be detected beyond the level of the interface (12, 35). Our findings emphasize the importance of considering the whole spectrum of user experience in both design and evaluation of digital health technologies. In particular, usability testing, which focuses mainly on the interface and task spheres, should be combined with other evaluation strategies, such as field deployments (46), that capture user experiences from real use in naturalistic settings, beyond individuals' interactions with the user interface.

### Competence Satisfaction and Frustration Most Prominent

The influence of the digital health technology on competence, in comparison to autonomy and relatedness, was most prominent in our data. Two themes, in particular, distinguished themselves in terms of typicality. *Increased knowledge and understanding* and *Increased awareness of health parameters* were clearly the most commonly occurring themes in our data. Respondents consistently reported an increased level of awareness and understanding about their behavior and lifestyle that created opportunities for developing their self-care skills. These experiences were mainly related to the provision of visual feedback, which belongs to the most common motivational affordances of persuasive technologies (47), and has been related to intentions to continued technology use (45). These examples of competence satisfaction may also be closely linked to an increased sense of autonomy, and are likely also promoted by autonomy-supportive strategies, such as provision of relevant information and a meaningful rationale for change (43). As highlighted in the SDT model of health behavior change, autonomy-support influences all three psychological needs and the satisfaction of competence is facilitated by autonomy (43). Thus, it should be considered that our examples reflecting competence satisfaction may also include experiences of autonomy satisfaction.

While competence satisfaction dominated in our data, we also found some indications of competence frustration. Some individuals described experiences of discouragement due to failure, which has been described as an unintended side effect of health behavior change support systems using gamification (32). The most common theme of competence frustration in our data concerned technical problems such as sync issues between the monitoring devices and the digital health app, non-functioning activity bracelets and poor perceived quality of monitored data (e.g., tracked number of steps not aligned with personal experiences). With reference to previous research on negative impacts of gamification, the technical problems can be understood as limiting issues that are related to unsuccessful implementation of features (36). One way to interpret the competence frustration resulting from technical problems is that, when interacting with technology, we may need to consider that the satisfaction of needs may be hierarchical. Hereby we mean that some basic needs may need to be satisfied before other needs can be supported. For example, in our study, usability challenges on the interface level may have prevented (some) users to experience the satisfaction or frustration of other needs with respect to using the digital health technology. If the technology does not work as intended or if a user does not know how to use it, its potential influence on psychological needs cannot be explored. Thus, to some extent, the satisfaction of competence needs on the interface and task levels may be a prerequisite for other needs to be supported by the technology. This may be a partial explanation for the relative scarcity of data reflecting the influence on the psychological need of autonomy in particular. However, as pointed out above, the satisfaction of autonomy and competence are likely linked, such that the satisfaction or frustration of one need may influence the satisfaction or frustration of other needs. Another possible explanation is that the design features of the digital health technology that was used in this study were more supportive of competence than autonomy and relatedness. Autonomy support can be provided by personalization and tailoring to enable users to tailor the technology to their individual needs, such as defining which health parameters to monitor and set their own aspired health goals (e.g., a physical activity target) (35, 37). The digital health technology that was used in this study contained some tailoring features that allowed users to customize their personal profile in terms of health goals, personal preferences and social data. However, based on the questionnaire data alone, we could not identify any impact on perceived autonomy.

### Tensions Between Satisfaction and Frustration of Psychological Needs

Our data revealed tensions in the satisfaction and frustration of all three psychological needs. The discussion has already touched upon tensions related to autonomy (autonomous vs. controlled behavior regulation) and competence (increased awareness, knowledge and understanding vs. discouragement as a result of failure). With respect to relatedness, some felt that the digital health technology made it easier for them to get and keep contact with healthcare, while others experienced frustration about digital communication, mainly due to the experience of insufficient or lacking responses from healthcare to expressed needs. Similarly, while some experienced healthcare staff as responsive to their behavior changes, others were frustrated by what they experienced as poor support or even disturbing use of external praise and rewards for accomplishments. Self-monitoring technologies, in particular, have raised polarized discussions, highlighting how it may either contribute to strengthen individual's autonomy or, conversely, contribute to increased control and surveillance over individuals (48).

We believe that the tensions in our data may be explained by individual variations in user profiles and preferences. Insufficient fit of the technology to the context and target users, and unsatisfactory interaction design (e.g., exaggerated feedback) have been identified as primary issues that may lead to negative experiences (49). It has also been shown that the use of tailoring, and personalization in particular, may lead to both positive and negative experiences (50). Previous research has found that the value of different design features may depend, for example, on variations in goal profiles (10). Gamification for the provision of feedback and external rewards suits individuals who are outcomes-focused, rather than focused on the process that leads to aspired outcomes. Similarly, quantified-self features may provide feedback that supports goal-setting, as well as evaluation of progress and behavior outcomes (51). However, users with less specified goals may not find quantified-self features as useful, perhaps because the actionability of data they receive through these features is low. Social networking features suit individuals who validate their performance based on comparison with others, while social networking does not fit individuals who have a tendency to avoid setting goals (10). Thus, even if the psychological needs are generic, there may be individual variations that may be addressed, for example by adequate tailoring. While a number of different tailoring design concepts have been described, it has also been emphasized that the use of these to promote healthy behaviors (e.g., through physical activity coaching) is still in its infancy (52). It has also been highlighted that the user profiles of older adults, in particular, have been given limited attention in the gamification research domain (53).

### Limitations

We acknowledge that our study has several limitations. We focused on individuals' perceptions of using a digital health service after it had been adopted and used for ~7 months. When studying this type of technological services, it is not always evident what behaviors and experiences are related to technology use as opposed to other factors. The METUX model was useful to separate user experiences into different spheres, although some experiences were challenging to classify. For example, are all self-monitoring activities that are supported by a digital health technology to be regarded as technology-specific tasks or should some of them be viewed as self-care behavior beyond technology use? For example, individuals may monitor their blood pressure, sleep or weight irrespective of using a particular health technology. However, any possible misclassifications will not have affected our results in terms of the identified themes of need satisfaction and need frustration that can be triggered by digital health technologies. Two spheres of user experience that are also described in the METUX model were not captured in our study: adoption and society (12). While we identified some reflections about the influence on needs on a society level, these were not descriptive enough in detail to classify which type of need they could be related to. Potential societal benefits in terms of public health, healthcare spending and productivity would be of value to explore further in future studies.

We acknowledge that the distinction between need frustration as opposed to low levels of need satisfaction was not always evident in our data. In SDT, satisfaction and frustration of basic needs are considered as two independent and asymmetrical dimensions (18, 19). Whereas need frustration by definition involves low need satisfaction, the opposite is not necessarily true. Need frustration can be described as an active and direct act in contrast with low levels of need satisfaction that are more passive and indirect (19). The distinction is important because it has been shown that experiences of need frustration are more predictive of illbeing (20). We believe that some of the examples in our data may clearly describe experiences of need frustration (e.g., *feeling pressured to perform self-monitoring)*, whereas others may in fact represent low levels of need satisfaction rather than need frustration (e.g., *insufficient quality of measurement results*). Thus, our results should be viewed as indicative, giving rise to hypotheses that need further exploration, preferably in combination with health and well-being outcomes data.

A recent review of motivational information systems discussed several future research trajectories, such as paying attention to different types of feedback, design features, and their effects on users, and the need to further take into account individual user attributes (13). Gamification design principles and a list of relating research questions for future research have also been proposed (54). We acknowledge that our study does not allow us to draw generalizable conclusions about the impact of individual design features on the satisfaction or frustration of psychological needs. While an in-depth study of individual design features and their effects was beyond the scope of our study, we may nevertheless suggest some general design considerations that may be applicable in similar contexts. Need satisfaction in our data was most prominent in relation to two of the main functional areas in the studied digital health technology: first, the visual feedback of self-monitored health parameters that was associated with competence satisfaction; and second, the communication feature that improved patients' access to healthcare staff and was associated with relatedness satisfaction. Thus, based on our study population, we suggest that functionalities for visual feedback of health parameters and chat/video communication with staff may be central need-supportive design features in digital health technologies supporting self-care in chronic care management. However, as discussed above, automated messages and nudges triggered both satisfaction and frustration. Therefore, we suggest that the value of personalization features and how to successfully design them should be explored further, ideally in relation to individual user attributes. Finally, as our results indicate that the shifting of health monitoring tasks traditionally performed by healthcare staff to patients may lead to both satisfaction and frustration of autonomy, we suggest that the design of self-monitoring technologies should provide tailoring that allows users to control at least the frequency of self-monitoring, and enable full privacy and control over their own health data (55), to prevent individuals from feeling controlled and monitored by healthcare or a third party.

The study was exploratory in nature and used survey responses to open-ended questions for a deductive thematic analysis. It has been argued that qualitative studies are well-suited to identify the manifestation of need satisfaction and frustration in individuals' narratives (18). The use of questionnaire data in qualitative research has been questioned as it is deemed difficult to fulfill excellence criteria for qualitative research with such data (56). Meeting these criteria requires timely and relevant research questions and findings, as well as rich and appropriate data (57). The research should “meaningfully interconnect literature, research questions/foci, findings, and interpretations with each other” (57). The data included in this study was purposefully gathered with the research question in mind, as part of a larger project exploring how individuals engaged in self-care experience the support from both healthcare and digital health technologies (unpublished). The data included 642 open-ended responses from 122 respondents. Many responses were elaborate and detailed, as showed in the citations presented in the findings. Nevertheless, we acknowledge the limitations at hand, such as the lack of opportunity to probe respondents for further details, which resulted in the exclusion of a number of items in our dataset that could not be used in the analysis. Thus, other means of data collection and analysis, such as individual interviews, possibly combined with data analytics on users' interactions with the digital health technology and behavior over time, would have provided more depth to our data and results. The strength of this qualitative study lies in its coverage, rather than depth. Nevertheless, we suggest that our analysis enriches the understanding of the phenomenon explored and that the data is appropriate for answering the a priori determined research question. In line with Braun et al. (58), we argue that qualitative survey questions can produce the rich accounts of the type of sensemaking typically explored using more common qualitative research methods such as interviews.

Given the limitations that have been addressed, we believe that we can motivate transferability of our findings to the field of persuasive technology focusing on quantified-self in chronic care management (9). While our sample covered individuals with some of the most common chronic conditions (cardiovascular diseases and mental illness) affecting public health, contextual factors, including the study setting and the digital health technology itself, need to be considered when transferring our findings to other settings. The design of gamification and social networking features, which are common in commercial apps promoting a healthy lifestyle, was less explored in our study. In contrast to these types of commercial digital health technologies, the use of digital health technologies in a healthcare setting involves a transition of care activities from healthcare staff to patients, which may entail a number of ethical dilemmas, such as accountability, intelligibility, and accessibility issues (33). One purpose of using digital health technologies in a healthcare setting is to enhance quality of care while limiting costs, which should be taken into consideration when discussing and comparing different types of digital health technologies and the setting they are used in. Our aim was not to evaluate specific design features, but rather to gain a general understanding of the satisfaction and frustration of psychological needs that a typical digital health technology that aims to support self-care in chronic care management can evoke. Nevertheless, we hope that our findings may trigger further research into exploring design issues in more depth.

## Conclusion

Our study contributes to the field of persuasive technology, in particular in relation to motivational design affordances based on quantified-self. We have focused on the exploration of the satisfaction and frustration of psychological needs that determine autonomous motivation. Based on theory, we know that the satisfaction of psychological needs is central to the maintenance of target behaviors and well-being. To develop digital health technologies that satisfy these needs, designers need to take into account these needs not only at the technology interface level, but at higher levels of user experience. We have demonstrated that engagement with digital health technologies in self-care may influence users in both positive and negative ways. This emphasizes the importance of being aware of the possible variability of goal profiles or other factors among users that may not all be equally compatible with different design features. Therefore, digital health technologies need to be flexible enough to accommodate for variation of user profiles. Future research should further explore variations in user profiles and how to design for flexibility. We believe that careful design considerations that take motivational theory into account will be necessary to transform care for individuals with chronic conditions.

## Data Availability Statement

The raw data supporting the conclusions of this article will be made available by the authors, without undue reservation.

## Ethics Statement

The studies involving human participants were reviewed and approved by the Regional Ethical Review Board of Stockholm (reg nr. 2018-1717-32), Tomtebodavägen 18A, 171 77 Stockholm, Sweden. The patients/participants provided their written informed consent to participate in this study.

## Author Contributions

CW and UvT designed the study and collected data, in collaboration with research colleagues acknowledged below. CW was mainly responsible for data analysis. CW, TS, and UvT drafted the manuscript. AS was expert on the theory that was applied, contributed with critical revision of the manuscript, and contribution of important intellectual content. All authors contributed to the interpretation of data, approved the final version of the manuscript, and agree to be accountable for all aspects of the work.

## Conflict of Interest

The authors declare that the research was conducted in the absence of any commercial or financial relationships that could be construed as a potential conflict of interest.
